# Development of Multiple Subcutaneous Lipomas After Six Months of Hormone Replacement Therapy: A Case Report

**DOI:** 10.7759/cureus.80551

**Published:** 2025-03-14

**Authors:** Kazuyoshi Iijima, Yoshihito Mima

**Affiliations:** 1 Department of Dermatology, Teikyo University Mizonokuchi Hospital, Kawasaki, JPN; 2 Department of Dermatology, Tokyo Metropolitan Police Hospital, Tokyo, JPN

**Keywords:** estrogen, hormone replacement therapy, lipomatosis, multiple lipomas, vegf

## Abstract

Lipomas are common benign soft tissue tumors composed of mature adipocytes, typically presenting as slow-growing, painless subcutaneous nodules. While the underlying etiology remains unclear, various factors have been proposed as potential contributors. In some individuals, multiple subcutaneous lipomas can develop, and their occurrence has been considered in relation to systemic conditions or familial predisposition. Hormone replacement therapy (HRT), commonly used for menopausal symptom management, influences lipid metabolism and angiogenesis through hormonal signaling pathways; however, its potential role in lipoma formation remains uncertain. Herein, we present a case of a 57-year-old woman who developed multiple subcutaneous lipomas on the thighs and buttocks after initiating HRT. This case highlights a possible association between hormonal changes and lipoma formation, emphasizing the importance of further research into the mechanisms underlying adipose tissue growth.

## Introduction

Lipomas are common benign mesenchymal tumors composed of mature adipose tissue. They typically manifest as slow-growing, soft, painless subcutaneous nodules, most commonly on the trunk and upper extremities, though they can develop in any region containing adipose tissue [1]. Despite their prevalence, the precise etiology of lipoma formation remains unclear, with genetic predisposition, metabolic influences, and trauma proposed as contributing mechanisms [1].

While solitary lipomas are more common, multiple lipomas may arise sporadically or in association with conditions such as familial multiple lipomatosis [2]. Beyond genetic and metabolic influences, hormonal factors have also been implicated in adipose tissue growth. Estrogen, in particular, plays a crucial role in lipid metabolism, fat distribution, and angiogenesis through its interaction with estrogen receptors (ER)α and Erβ [3].

Hormone replacement therapy (HRT) is widely prescribed to postmenopausal women to alleviate symptoms of estrogen deficiency and prevent osteoporosis. Estrogen exerts its effects on adipose tissue primarily through ERs, modulating key factors such as vascular endothelial growth factor (VEGF) and lipoprotein lipase (LPL) [3]. These down-regulating pathways influence adipogenesis, lipid metabolism, and angiogenesis, providing a plausible mechanistic link between hormonal modulation and lipoma development.

Herein, we report a case of a 57-year-old woman who developed multiple subcutaneous lipomas, predominantly on the thighs and buttocks, following the initiation of HRT. This report suggests the potential role of estrogen-mediated pathways in lipoma formation, emphasizing the need for further research into the hormonal regulation of adipose tissue proliferation.

## Case presentation

**Figure 1:**
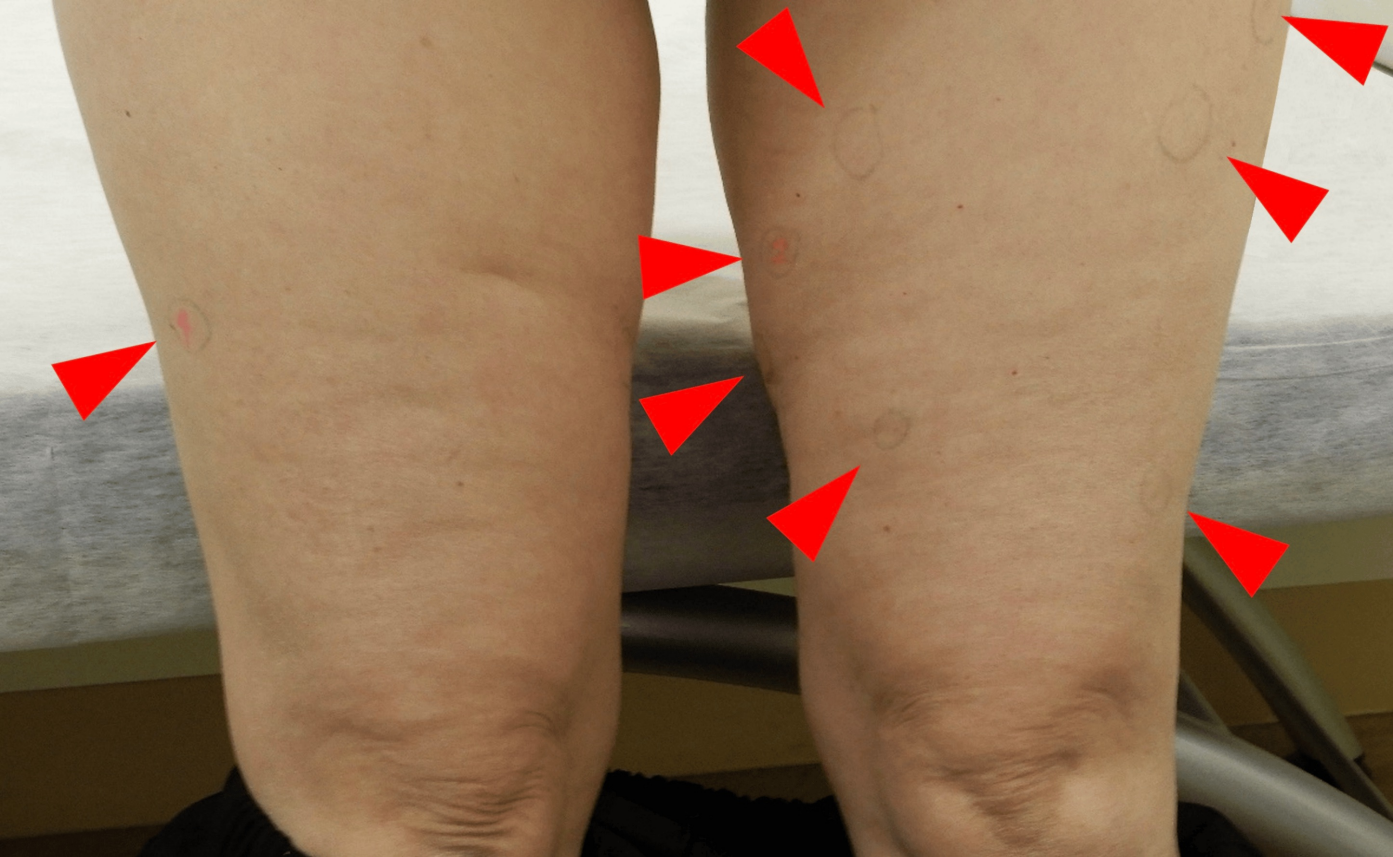
Initial clinical photograph showing multiple subcutaneous lipomas in the thigh (arrowheads).

**Figure 2:**
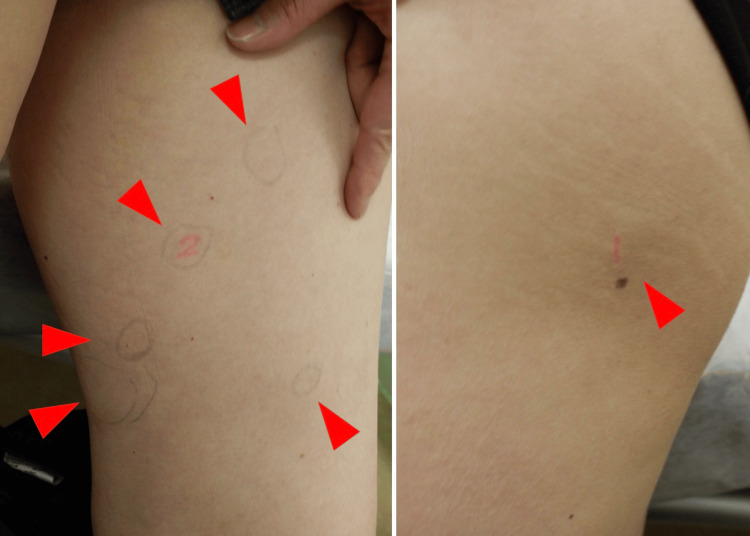
Clinical photographs showing multiple subcutaneous lipomas in the left inner and upper thigh (arrowheads).

A 57-year-old woman presented with multiple subcutaneous nodules that had developed progressively over the past 18 months. She had a medical history of dyslipidemia, for which she had been taking atorvastatin for the past 10 years. Two years prior to presentation, estrogen and progesterone therapy was initiated for the management of menopausal symptoms. Approximately six months after starting HRT, she noticed the subcutaneous masses, predominantly on the thighs and buttocks, which had gradually been enlarging, prompting further evaluation. The patient had no family history of similar lesions. She denied spontaneous pain or tenderness. Laboratory examination, including collagen disease markers, thyroid function tests, liver and renal function tests, and lipid profiles, revealed no abnormalities. Physical examination revealed multiple subcutaneous nodules (10 in total) on the thighs and buttocks (Figures 1, 2).

The lesions were well-defined, soft to firm in consistency, with smooth surfaces and no overlying skin discoloration. No signs of inflammation or tenderness were noted.

**Figure 3:**
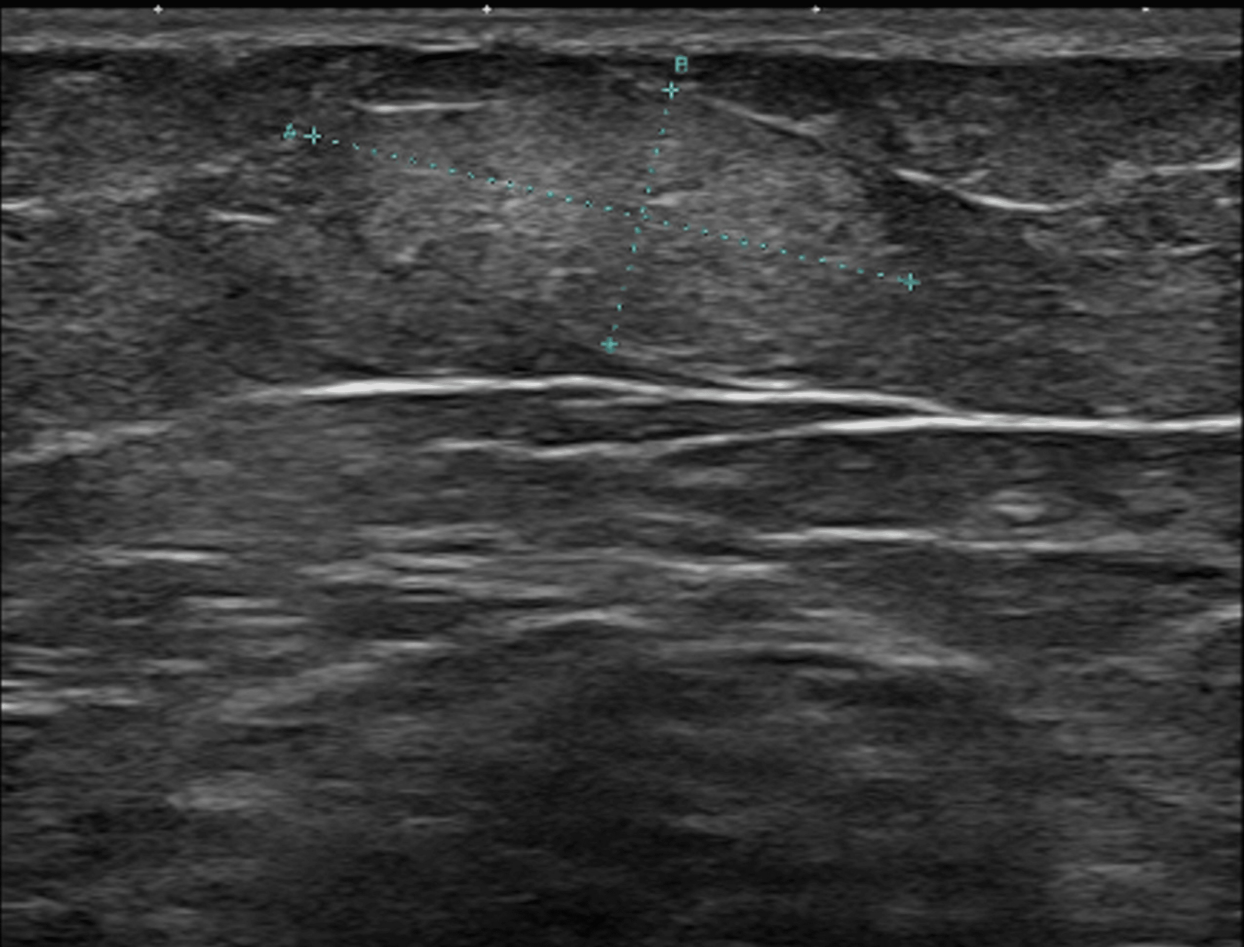
Ultrasound image of a subcutaneous lipoma measuring 1.4 × 2.0 cm. The lesion is well-circumscribed and hypoechoic, with internal echogenic strands, consistent with the typical sonographic appearance of a lipoma.

Ultrasonography revealed multiple well-circumscribed hypoechoic lesions within the subcutaneous fat layer, ranging in size from 0.5 cm to 2.0 cm in maximum diameter, including a representative lesion measuring 1.4 × 2.0 cm, shown in Figure 3.

The patient continued HRT and reported a slight subjective increase in the size of some lipomas over time; however, no significant changes were observed on clinical evaluation.

## Discussion

Lipomas are the most common benign tumors of adipose tissue, characterized by slow growth and a well-encapsulated structure. They primarily develop in the subcutaneous layer but can occasionally arise in deeper structures or visceral organs. These tumors are typically soft, mobile, and painless, often discovered incidentally due to their asymptomatic nature.

While solitary lipomas are the most prevalent, multiple subcutaneous lipomas occur in approximately 5-15% of cases [4]. Rydholm and Berg reported that among 428 cases of non-visceral lipomas diagnosed within one year, 61 (14%) involved multiple lipomas, with a significantly higher prevalence in men [2]. These lipomas may arise sporadically or be associated with genetic conditions such as familial multiple lipomatosis. Although benign, multiple lipomas can be particularly burdensome for patients, especially when widespread. In some cases, large lipomas may cause compressive symptoms, necessitating medical intervention.

The pathogenesis of lipomas remains unknown, with trauma, metabolic dysregulation, and genetic predisposition being proposed as contributing factors [1]. Several studies have explored the link between blunt trauma and lipoma formation, proposing two potential mechanisms: (1) direct mechanical impact leading to adipose tissue herniation through the fascia and (2) cytokine-mediated preadipocyte differentiation and proliferation following soft tissue trauma [5,6].

In addition to mechanical factors, metabolic disturbances, particularly lipid abnormalities, have been implicated in lipoma development. Rubinstein et al. reported that in families with familial combined hyperlipidemia, non-symmetric subcutaneous lipomas were exclusively present in affected individuals, with a positive correlation between hyperlipidemia severity and lipoma count [7]. Similarly, Dawoud et al. described symmetrical subcutaneous lipomatosis in two pediatric patients with familial hypercholesterolemia [8], further suggesting the role of dyslipidemia in adipose tissue proliferation.

Genetic influences are evident in several conditions associated with multiple lipomas, including familial multiple lipomatosis, angiolipomatosis, Dercum’s disease, and multiple symmetric lipomatosis (Madelung’s disease). Additionally, lipomas [9] and angiolipomas [10] have been reported following anabolic steroid use, and platinum-based chemotherapy has been associated with visceral multiple lipomas [11], suggesting potential hormonal or pharmacological triggers.

HRT is widely used to manage postmenopausal symptoms by supplementing estrogen and progesterone. Estrogen exerts diverse physiological effects, including regulation of bone density, cardiovascular function, and lipid metabolism, while also influencing fat distribution. In the present case, a 57-year-old woman developed multiple lipomas on the thighs and buttocks six months after initiating HRT, with a tendency for further lipoma formation continuing in parallel with ongoing HRT, which raised questions regarding the role of estrogen in lipoma formation.

Estrogen regulates adipose tissue metabolism through ERα and ERβ, with ERα being particularly involved in adipocyte differentiation and lipid homeostasis [3]. Clinical studies have demonstrated that estrogen promotes subcutaneous fat accumulation, particularly in the thighs and buttocks, whereas its decline after menopause shifts fat deposition toward the visceral compartment [12,13]. However, the precise effects of estrogen on fat accumulation remain inconsistent, varying by species, fat depot location, and experimental methodologies [3].

Beyond its direct effects on adipocytes, estrogen may also indirectly influence lipoma formation through its roles in lipid metabolism and angiogenesis. LPL, a key enzyme in lipid storage, is regulated by estrogen [3]. However, its effects on LPL activity in humans remain controversial, with studies reporting both increased and decreased activity depending on the adipose depot and experimental conditions [14,15]. Park et al. demonstrated that lipomas exhibit increased heparin-releasable LPL activity compared to surrounding normal adipose tissue and a higher proportion of a partially glycosylated, 55kDa LPL isoform [16]. These findings suggest that alterations in LPL expression and glycosylation may contribute to lipoma development. As estrogen modulates lipid metabolism and LPL activity in a depot- and condition-dependent manner, HRT-related estrogen supplementation may influence these pathways, potentially affecting lipoma formation and growth. Additionally, Prakash et al. reported an association between hypertriglyceridemia and lipoma formation [1]. Estrogen has been shown to influence hepatic very low-density lipoprotein (VLDL) production, which in some cases leads to elevated plasma triglyceride levels, a recognized effect of HRT. However, the relationship between systemic lipid alterations and local LPL activity in lipoma development remains unclear. It is possible that changes in triglyceride metabolism modulate LPL activity within adipose tissue, thereby affecting lipoma formation. Further research is needed to elucidate the specific mechanisms underlying these interactions.

Apart from its role in lipid metabolism, estrogen has also been implicated in angiogenesis. Estrogen induce VEGF expression via ERα activation [17]. Both VEGF and epidermal growth factor receptor (EGFR) have been implicated in lipoma development [18]. Nakao et al. further suggested that neurovascular perforators and mechanical stimulation may contribute to lipoma formation [19]. Thus, estrogen may promote vascular remodeling and adipocyte expansion, potentially supporting lipoma formation and progression.

Lee et al. examined the expression of ER, progesterone receptor (PR), VEGF, and EGFR in lipoma tissue. Their findings indicated that ER and PR positivity was significantly lower, suggesting a limited role for these hormones in lipoma development. In contrast, VEGF expression was markedly elevated, indicating the potential role of angiogenesis in tumor growth. Additionally, some lipoma cases exhibited EGFR positivity, highlighting a possible but yet undefined involvement of EGFR signaling in lipoma formation. These findings suggest that angiogenic pathways may play a more prominent role than hormonal signaling in lipoma pathogenesis, warranting further investigation [20]. From a clinical perspective, asymptomatic lipomas are typically observed without intervention, while symptomatic or cosmetically concerning cases may require surgical excision. Further studies are needed to explore potential treatment options for cases where surgical intervention is not preferred.

## Conclusions

In the present case, a 57-year-old woman developed lipomas on the thighs and buttocks six months after initiating HRT, with a tendency for further lipoma formation as HRT continued. This suggests a potential association between HRT administration and the development of multiple lipomas. Estrogen may contribute to lipoma formation, not only by directly influencing adipocyte metabolism and differentiation, but also indirectly through vascular remodeling and adipocyte proliferation via ERα-mediated VEGF expression. However, the effects of estrogen remain controversial, varying depending on experimental conditions, and its precise role in lipoma formation remains unclear. Given that many lipomas are asymptomatic and often go unreported, the true prevalence of HRT-associated lipoma formation may be underestimated. While this report suggests a possible link between HRT and lipoma formation, the absence of histopathological and molecular analyses remains a limitation. Further research incorporating detailed tissue and biochemical investigations will be essential to elucidate the underlying mechanisms and the role of hormonal modulation in adipose tissue growth.
